# Predicting Severe/Critical Outcomes in Patients With SARS-CoV2 Pneumonia: Development of the prediCtion seveRe/crItical ouTcome in COVID-19 (CRITIC) Model

**DOI:** 10.3389/fmed.2021.695195

**Published:** 2021-09-08

**Authors:** Fausto Salaffi, Marina Carotti, Marco Di Carlo, Luca Ceccarelli, Massimo Galli, Piercarlo Sarzi-Puttini, Andrea Giovagnoni

**Affiliations:** ^1^Rheumatology Clinic, Dipartimento di Scienze Cliniche e Molecolari, Università Politecnica delle Marche, Jesi, Italy; ^2^Dipartimento di Scienze Radiologiche Struttura Organizzativa Dipartimentale Radiologia Pediatrica e Specialistica, Azienda Ospedaliera Universitaria, Ospedali Riuniti di Ancona, Ancona, Italy; ^3^Unità Operativa di Radiologia – Ospedale degli Infermi, Azienda Unità Sanitaria Locale della Romagna, Faenza, Italy; ^4^Divisione di Malattie Infettive, Dipartimento di Scienze Biomediche e Cliniche “Luigi Sacco, ” Azienda Socio Sanitaria Territoriale Fatebenefratelli-Sacco, Milan University School of Medicine, Milan, Italy; ^5^Divisione di Reumatologia, Dipartimento di Scienze Biomediche e Cliniche “Luigi Sacco, ” Azienda Socio Sanitaria Territoriale Fatebenefratelli-Sacco, Milan University School of Medicine, Milan, Italy

**Keywords:** COVID-19, prediction model, Charlson Comorbidity Index, age, lung computed tomography

## Abstract

**Objective:** To create a prediction model of the risk of severe/critical disease in patients with Coronavirus disease (COVID-19).

**Methods:** Clinical, laboratory, and lung computed tomography (CT) severity score were collected from patients admitted for COVID-19 pneumonia and considered as independent variables for the risk of severe/critical disease in a logistic regression analysis. The discriminative properties of the variables were analyzed through the area under the receiver operating characteristic curve analysis and included in a prediction model based on Fagan's nomogram to calculate the post-test probability of severe/critical disease. All analyses were conducted using Medcalc (version 19.0, MedCalc Software, Ostend, Belgium).

**Results:** One hundred seventy-one patients with COVID-19 pneumonia, including 37 severe/critical cases (21.6%) and 134 mild/moderate cases were evaluated. Among all the analyzed variables, Charlson Comorbidity Index (CCI) was that with the highest relative importance (*p* = 0.0001), followed by CT severity score (*p* = 0.0002), and age (*p* = 0.0009). The optimal cut-off points for the predictive variables resulted: 3 for CCI [sensitivity 83.8%, specificity 69.6%, positive likelihood ratio (+LR) 2.76], 69.9 for age (sensitivity 94.6%, specificity 68.1, +LR 2.97), and 53 for CT severity score (sensitivity 64.9%, specificity 84.4%, +LR 4.17).

**Conclusion:** The nomogram including CCI, age, and CT severity score, may be used to stratify patients with COVID-19 pneumonia.

## Introduction

Coronavirus disease (COVID-19) is a highly life-threatening infectious disease. The pandemic of the novel severe acute respiratory syndrome coronavirus 2 (SARS-CoV-2) pandemic, which is still ongoing, has resulted in huge costs in terms of human lives, economic and social damage (1, 2). COVID-19 pneumonia can result in acute respiratory distress syndrome (ARDS) and multiple organ failure (3, 4). The severe/critical patients have a poor prognosis and a high mortality rate as compared to the mild/typical patients (5, 6). In patients admitted to hospitals have been identified certain factors related to increased severity (7, 8).

The use of imaging in the diagnosis and identification of risk factors for the development of COVID-19 pneumonia is important. Lung CT is the first-line imaging modality in cases where there is a deep suspicion (9). CT can accurately evaluate the type and extent of lung lesions (10). Recent studies showed that lung CT may enable the detection of the disease with higher sensitivity in comparison to reverse-transcription polymerase chain reaction (RT-PCR). A recent meta-analysis performed to determine the diagnostic accuracy of the initial lung CT scan compared to RT-PCR in COVID-19 patients, demonstrated that the sensitivity of CT, compared to RT-PCR, was 87% and that the specificity was 46%, with a positive predictive value of 69% and a negative predictive value of 89% (11). Although lung CT findings such as consolidation, linear opacities, crazy-paving pattern, bronchial wall thickening, and extrapulmonary lesions are features of severe/critical COVID-19 pneumonia (10), there is still limited information about the prognostic implication of CT findings in patients with COVID-19. Identifying admission CT predictors of adverse outcome in patients without underlying medical issues would help to identify the most vulnerable patients in this age range and, as a result, change their therapy. Several recent studies reported imaging CT findings of patients with adverse outcomes (12–15). However, data on the outcomes of consecutive patients, factors influencing hospital admission vs. outpatient management, and risk factors for COVID-19 adverse effects is still lacking in Italy. The significance of recognizing patients that are at risk of developing severe COVID-19 is mandatory for optimizing intra-hospital resources. Patients with serious illnesses often need a wide range of medical services. As a result, through patient prioritization, early identification of patients at high risk of developing severe COVID-19 will encourage appropriate supportive treatment and reduce mortality rates, as well as unnecessary or insufficient healthcare usage (16, 17).

As a method for rapid clinical management of hospitalized patients, the aim of this study is to build an individualized research model to identify the risk of severe/critical disease in patients with COVID-19 pneumonia.

## Methods

### Study Design and Participants

The present study is an extension of the data collected in a previous study aimed at realizing a prognostic lung CT score for worse outcomes in COVID-19 pneumonia (18). In that study, between February 20th, 2020 and April 15th, 2020, data concerning a cohort of patients diagnosed as having COVID-19 pneumonia were retrospectively collected from four hospitals in the Italian regions of Marche, Lombardy and Emilia-Romagna. The inclusion criteria were: an epidemiological history plausible of SARS-CoV2 infection, the identification of SARS-CoV-2 nucleic acid in throat swabs or the lower respiratory tract by real-time reverse transcription polymerase chain reaction, and at least one thin-section lung CT. One the basis of the clinical stages of COVID-19 proposed by WHO (19), patients were assigned to 1 of 2 categories: those with mild/moderate disease and those with severe/critical disease. Mild symptomatic patients meeting the case definition for COVID-19 without evidence of viral pneumonia or hypoxia. Moderate patients meeting clinical signs of pneumonia (fever, cough, dyspnea, and fast breathing) but no signs of severe pneumonia, including pulse oximeter oxygen saturation ≥90% on room air. Severe disease was defined a respiratory rate of ≥30 beats per minute, or ≤ 93% resting oxygen saturation, or arterial oxygen partial pressure (PaO_2_)/fraction of inspired oxygen (FiO_2_) ≤ 300 mmHg (1 mm Hg = 0.133 kPa), or a ≥50% progression of lung CT findings of pneumonia (fever, cough, dyspnea, fast breathing) within 24–48 h (20). Critical disease was defined as admission to an ICU for mechanical ventilation or oxygenation impairment (mild ARDS: 200 mmHg < PaO_2_/FiO_2_ ≤ 300 mmHg [with positive end-respiratory pressure (PEEP) or continuous positive airway pressure ≥5 cmH_2_O]; moderate ARDS: 100 mmHg < PaO_2_/FiO_2_ ≤ 200 mmHg (with PEEP ≥5 cmH_2_O); severe ARDS: PaO_2_/FiO_2_ ≤ 100 mmHg (with PEEP ≥5cmH_2_O) (21). The patients' recorded demographic and clinical characteristics included age and sex, the time since symptom onset to hospital admission, co-morbidities (systemic hypertension, diabetes mellitus, heart disease, and chronic obstructive pulmonary disease), symptoms, and clinical and laboratory signs.

### Predictors

Several variables were collected from the electronic health record: age at time of testing, sex, height, weight, dyspnea, days from illness onset, smoking history, and comorbidity burden.

Laboratory values were analyzed for the “worst” value occurring during the day of admission for inpatients. For the purposes of this study only C-reactive protein (CRP) was collected as laboratory variable.

All patients had lung CT scans, which were examined by two radiologists who had no access to the clinical or laboratory results. We selected these predictors based on previous published literature and our clinical experience of patients with COVID-19 (18, 22).

Dyspnea was assessed with the Borg Dyspnea Index (Borg score) (23). The Borg score evaluates the perceived dyspnea (breathing discomfort) with a numerical rating scale from 0 to 10 [0 = no breathlessness at all, 0.5 = very very slight (just noticeable), 1 = very slight, 2 = slight breathlessness, 3 = moderate, 4 = somewhat severe, 5 = severe breathlessness, 7 = very severe breathlessness, 9 = very, very severe (almost maximum) and 10 = maximum].

The Charlson Comorbidity Index (CCI) estimated the comorbidity burden (24). The CCI includes the following comorbid conditions: acute myocardial infarction, congestive heart failure, peripheral vascular disease, cerebrovascular disease, dementia, chronic obstructive pulmonary disease, rheumatoid arthritis, peptic ulcer disease, mild and moderate/severe liver disease, diabetes mellitus with and without complications, hemiplegia/paraplegia, renal disease, cancer (any malignancy) and metastatic solid tumor, AIDS/HIV. When computing the weighted CCI, each condition from the CCI is given a ranking, regardless of the coding system used. In particular, diabetes with complications, hemiplegia/paraplegia, renal disease, and malignancies are assigned a score of 2; moderate/severe liver disease is assigned a score of 3; metastatic solid tumor and AIDS/HIV are assigned a score of 6; the remaining comorbidities are assigned a score of 1. The total score in the CCI is derived by summing the assigned weights of all comorbid conditions. Higher scores indicate a more severe condition and consequently, a worse prognosis. While it was initially designed to predict mortality risk after hospitalization, it has been shown to predict adverse outcomes independently over a wide range of conditions (25).

### Lung Computed Tomography Analysis

Two CT scanners with helical acquisitions in end-inspiration were used for the lung CT examinations. The detailed protocol of CT image acquisition is described elsewhere (18).

Lung CT examinations were independently examined by two radiologists with 3 (LC) and 20 (MC) years of lung CT interpretation experience, respectively. The images were viewed on both lung (width, 1,500 HU; level, −700 HU) and mediastinal (width, 350 HU; level, 40 HU) settings. The two readers analyzed the axial CT images but were free to evaluate the multiplanar reformats. The CT scans were assessed for the presence of ground-glass opacities, consolidation, cavitation, centrilobular nodules, tree-in-bud pattern, septal thickening, perilobular opacities, reticulation, architectural distortion, subpleural bands, traction bronchiectasis, bronchial wall thickening, intrathoracic lymph node enlargement, and pleural effusions. Ground-glass opacity (GGO) was defined as increased lung density with no obscuration of the underlying lung markings. Increased lung density with obscuration of the underlying lung markings was known as consolidation. Polygonal or curvilinear bands bordering the secondary pulmonary lobule were known as perilobular opacities. Interlobular or intralobular irregular septal thickening was known as reticulation. Thin linear opacities peripheral and parallel to the pleura were known as subpleural bands. Traction bronchiectasis was defined as irregular or distorted dilated airways seen in areas of fibrosis.

The degree and type of abnormalities were measured to determine the severity of pulmonary parenchymal involvement using a semi-quantitative scoring (19). In this scoring system, each lung was evaluated on three levels: upper (above the carina), middle (below the carina up to the upper limit of the inferior pulmonary vein), and lower (below the inferior pulmonary vein). Each level's final score was determined by combining the results of the right and left lungs. The percentage of lung involvement in each level was evaluated independently and categorized as follows: 0 as normal, 1 as <25% abnormality, 2 as 25–49% abnormality, 3 as 50–74% abnormality, and 4 as ≥75% of the pulmonary cross-section CT scan. Further, the scoring system included the nature of abnormalities on a 4-point scale for defining the pattern of CT lung abnormalities. The two scores (abnormality degree and nature) were then multiplied by each other. After adding the scores from all six levels (3 levels on each side), a final radiologic severity score for parenchymal involvement was measured for each patient, with values ranging from 0 to 96. The previous work recorded the inter-observer agreements between the two readers for the measurement of lung CT-severity scores were excellent, with an intraclass correlation coefficient (ICC) of 0.987 (95% CI. 0.982–0.991; *p* < 0.001). The detailed description of the CT scoring system is provided in the first study in which the method is proposed (18).

### Statistical Analysis

All analyses were conducted using Medcalc (version 19.0, MedCalc Software, Ostend, Belgium). Kolmogorov-Smirnov test was used to evaluate the normality of quantitative data. Mean and standard deviation (SD) were used to describe normally distributed data, while median and interquartile range (IQR) was used to describe non-normally distributed data. Categorical variables were presented as numbers and percentages. Student's *t*-test, Mann-Whitney *U*-test, Pearson χ^2^-test, and Fisher's exact test were used to compare variables between mild/moderate disease vs. severe/critical disease.

Logistic regression analysis was used to assess the factors linked to the risk of severe/critical disease. Covariates considered in the model included: age, gender, BMI, days from illness onset, CRP, smoking history, Borg score, CCI, and lung CT severity score. The results were expressed as multivariate coefficients with standard errors and Wald statistic for the number of variables entered in the analysis. Significance was set at *p* < 0.05.

Then a predictive model was realized based on Bayes' theorem for determining severe/critical disease (post-test probability) using the pre-test probability of disease and the product of the positive likelihood ratio (+LR) from the predictive variables. The Fagan nomogram was used to graphically represent the model. The predictor variables included in the predictive model were those that demonstrated significance at logistic regression analysis. The predictive performance of each variable was estimated by the area under the receiver operating characteristic curve (AUC-ROC). Youden's index on the ROC curve analysis was used to determine the optimal cut-off point for the single predictive variables (26).

## Results

### Patient Characteristics

The study involved 171 COVID-19 patients [136 men (79.5%) and 35 women (20.5%), mean age (SD) 61.9 (11.8) years, range 33–86 years]. Thirty-seven (21.6%) had severe/critical disease and 134 (78.4%) mild/typical disease. The mean time (SD) interval between the days from illness onset was 6.3 (4.9).

One hundred and fifty-one (88.3%) patients complained of fever, in the majority of the patients (120/171, 70.2%) >38°C. Dry cough was also a common symptom at onset (108/171, 63.15%), followed by fatigue (102/171, 59.64%), myalgia (86/171, 50.29%), anosmia (67/171, 39.18%), and dyspnea (70/171, 40.9%). The mean (SD) Borg score was 2.61 (1.57).

Comorbidities were detected in 89 (52%) patients, of whom 49.4% (44/89) had only one comorbidity. The mean (SD) CCI was 3.37 (2.21). Of note, severe/critical group had a higher frequency of comorbidities, especially more than one comorbidity (all *p* < 0.001), compared with mild/typical group). Compared with mild/typical group, patients in severe/critical group were older (*p* < 0.001) and more frequently males (*p* < 0.05). Ninety-four of these 171 (54.9%) patients were admitted to hospital of whom 57 (60.6%) were discharged alive without hospital care. Among the 37 patients admitted to hospital, with critical illness, 25 (67.6%) required mechanical ventilation, nine (24.3%) required intensive care without mechanical ventilation, and three patients (8.1%) died.

The most common pattern seen on lung CT was multiple lobe involvement (90.9%) with more extensive GGO (81.9%) than consolidation (71.3%). The patients with severe/critical disease had a higher prevalence of consolidation (*p* = 0.031), interlobular septal thickening (*p* = 0.005), a crazy-paving pattern (*p* = 0.023), reticular opacities (*p* = 0.009), and air bronchogram (*p* = 0.001). The mean (SD) lung CT-severity score was 76.65 (6.93) in the group with severe/critical disease and 61.69 (13.21) in the group with mild/typical disease (*p* = 0.001). Table 1 summarizes the differences between mild/moderate group and severe/critical group.

**Table 1:** Demographic, clinical, laboratory findings of patients on admission.

	**All**	**Mild/moderate disease**	**Severe/critical disease**	***p***
Total sample, N° (%)	171 (100%)	134 (78.47%)	37 (21.63%)	—
Age years, mean (SD)	64.91 (13.59)	61.69 (13.21)	76.65 (6.93)	0.001
Male patients, N° (%)	136 (79.53%)	102 (76.11%)	34 (91.89%)	0.021
Smoking history, N° (%)	39 (22.83%)	28 (20.89%)	11 (29.72%)	0.039
Body Mass Index, kg/m^2^, mean (SD)	25.58 (3.85)	24.31 (3.97)	27.55 (3.21)	0.041
Charlson Comorbidity Index, mean (SD)	3.37 (2.21)	2.72 (1.69)	5.72 (2.28)	0.001
Signs and symptoms on admission				
Days from first symptoms, mean (SD)	6.3 (4.92)	5.1 (3.92)	7.4 (3.22)	0.034
Fever ≥ 38.8°C, N° (%)	120 (70.21%)	93 (69.4%)	27 (74.41%)	0.176
Dry cough, N° (%)	108 (63.15%)	85 (63.4%)	23 (62.21%)	0.784
Fatigue, N° (%)	102 (59.64%)	79 (58.95%)	23 (62.16%)	0.482
Myalgia, N° (%)	86 (50.29%)	66 (49.25%)	20 (54.05%)	0.091
Anosmia, N° (%)	67 (39.18%)	51 (38.305%)	15 (40.54%)	0.599
Dyspnea, N° (%)	63 (36.84%)	47 (35.07%)	16 (43.24%)	0.058
Borg dyspnea index, mean (SD)	2.61 (1.57)	2.07 (1.61)	3.25 (1.44)	0.025
Laboratory findings				
C-Reactive protein, mg/L, mean (SD)	11.28 (13.75)	9.40 (13.65)	11.87 (14.26)	0.046
Computed tomography findings				
Ground-glass opacity, N° (%)	140 (81.87%)	110 (82.15%)	30 (81.11%)	0.768
Consolidation, N° (%)	122 (71.34%)	93 (69.41%)	29 (78.42%)	0.031
Interlobular septal thickening, N° (%)	78 (45.61%)	49 (36.56%)	29 (78.37%)	0.005
Reticular opacity, N° (%)	76 (44.44%)	50 (37.31%)	26 (70.27%)	0.009
Air bronchogram, N° (%)	69 (40.35%)	46 (34.32%)	23 (62.16%)	0.021
Crazy-paving pattern, N° (%)	35 (20.54%)	24 (17.91%)	11 (29.72%)	0.023
Lymph node enlargement, N° (%)	25 (14.61%)	20 (14.92%)	5 (13.51%)	0.679
Pericardial effusion, N° (%)	16 (9.35%)	11 (8.21%)	5 (13.51%)	0.066
Pleural effusion, N° (%)	14 (8.18%)	10 (7.46%)	4 (10.81%)	0.054
Lung computed tomography severity score, mean (SD)	64.91 (13.59)	61.69 (13.21)	76.65 (6.93)	0.001

*SD, standard deviation*.

### Variables Predicting a Severe/Critical Disease

In logistic regression model, only the variables found to be significant in the univariate analysis were considered in predicting a severe/critical disease. Among all the analyzed variables, CCI was found to have the highest relative importance with a Wald value of 15.99 (*p* < 0.0001), followed by CT severity score (Wald 13.41; *p* = 0.0002) and age (Wald 10.94; *p* = 0.0009) (Table 2).

**Table 2:** Logistic regression analysis of the dependent variables predicting the development of a severe/critical disease.

**Variable**	**Coefficient**	**Standard error**	**Wald**	***p***
Constant	−19.76394	4.59177	18.5262	<0.0001
Age (years)	0.14981	0.045292	10.9411	0.0009
Gender	−0.50030	0.94867	0.2781	0.5979
Smoking history	1.46950	0.85989	2.9205	0.0875
Body Mass Index	0.017179	0.10363	0.02748	0.8683
Days from illness onset	−0.082411	0.045339	3.3039	0.0691
C-reactive protein (mg/L)	−0.013485	0.030438	0.1963	0.6577
Borg score	0.16300	0.21696	0.5645	0.4525
Charlson Comorbidity Index	1.07359	0.26844	15.9954	0.0001
Lung computed tomography severity score	0.086068	0.023500	13.4143	0.0002

**Figure 1:**
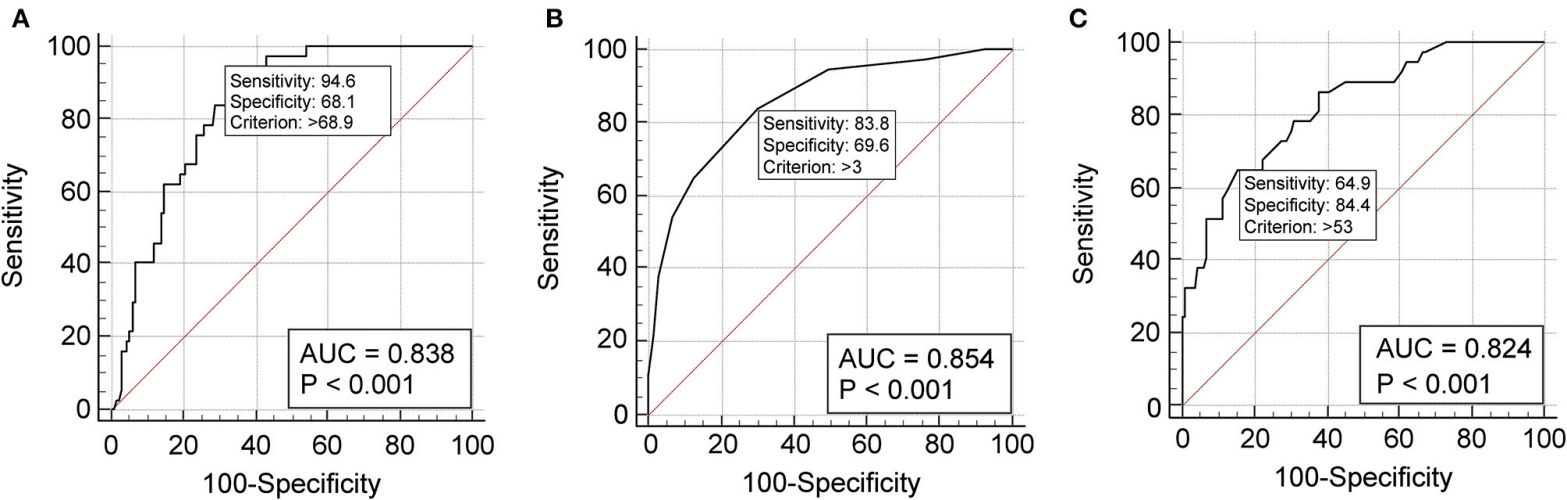
Receiver operating characteristic curve analysis showing the prognostic value of age **(A)**, Charlson Comorbidity Index **(B)**, and chest computed tomography severity score **(C)** in the discriminative performance for severe/critical disease. **(A)** The cut-off value of 68.9 years predicts a severe/critical disease with 94.6% sensitivity and 68.1% specificity. The AUC-ROC is 0.836 (95% CI 0.785–0.896), the Youden index 0.627; **(B)** the cut-off value of 3 at Charlson Comorbidity Index predicts a severe/critical outcome with 83.8% sensitivity and 69.6% specificity. The AUC-ROC curve is 0.854 (95% CI 0.792–0.903), the Youden index 0.534; **(C)** the cut-off value of 53 at computed tomography severity score predicts a severe/critical outcome with 64.9% sensitivity and 84.4% specificity. The AUC-ROC curve is 0.824 (95% CI 0.759–0.878), the Youden index 0.625. ROC, receiver operating characteristic; AUC, area under the curve; CI, confidence interval.

The predictive performance of a single variable was estimated by the area under the AUC-ROC curve analysis. Age showed an AUC of 0.838 (*p* = 0.0001) (Figure 1A) and an optimal cut-off point of 69.9 (sensitivity 94.6%, specificity 68.1%, +LR 2.97) (Supplementary Table 1). The CCI demonstrated excellent discriminative ability, with an AUC of 0.854 (*p* = 0.0001) (Figure 1B) and an optimal cut-off point of 3 (sensitivity 83.8%, specificity 69.6%, +LR 2.76) (Supplementary Table 2). The CT severity score revealed an AUC of 0.824 (*p* = 0.0001) (Figure 1C) and an optimal cut-off point of 53 (sensitivity 64.9%, specificity 84.4%, +LR 4.17) (Supplementary Table 3).

**Figure 2:**
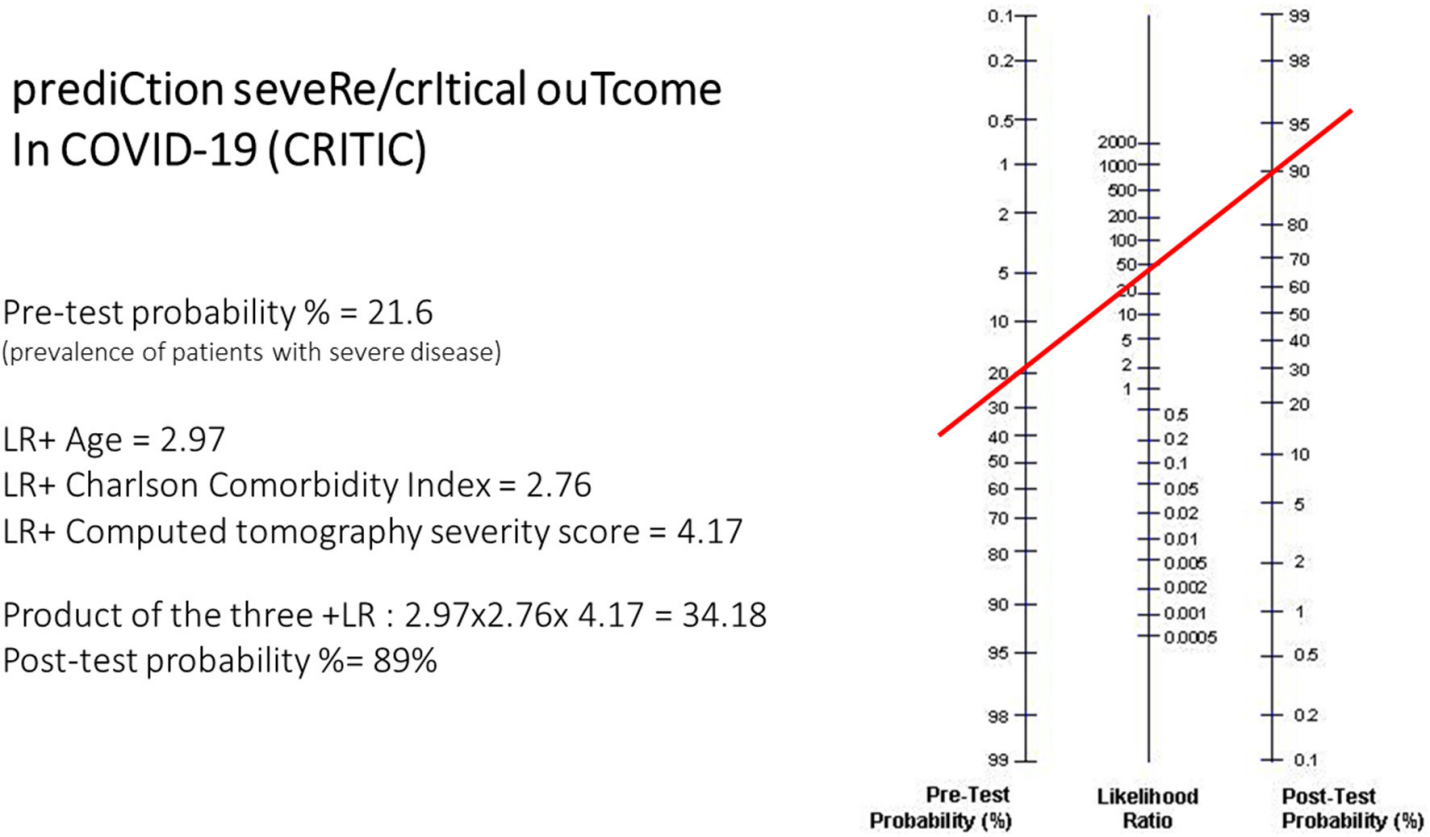
Example of application of the nomogram in the calculation of the CRITIC post-test probability. In the nomogram left axis represents the pre-test probability (21.6% in this case study), middle axis represents positive likelihood ratio, and the right axis shows post-test probability. To calculate the actual risk (post-test probability, %) of severe/critical disease in a given patient with COVID-19 pneumonia, the positive likelihood ratio of each item in that patient have to be multiplied. The resulting positive likelihood ratio product represents the point intercepted on the middle axis.

The three predictive variables (age, CCI, CT severity score) can be applied to the Fagan nomogram to calculate the post-test probability of severe/critical disease risk (Figure 2). Calculation of the post-test probability is based on the pre-test probability (21.6% in the case series studied) and the product of the +LRs of the three predictor variables in the individual patient. This type of calculation is suitable to be performed through smartphone apps. With the results of this study, a quick and user-friendly smartphone app based on these findings was created to calculate the post-test probability of severe/critical disease in COVID-19 pneumonia patients (CRITIC-App, Fausto Salaffi, 2021, all rights reserved; the app is accessible for iOS/Android mobile platforms to interested researchers at website http://www.faustosalaffi.it/app/covid/).

## Discussion

In this study it has been proposed a prediction model, calculated at hospital admission, to establish the subsequent presence of disease deterioration and the occurrence of severe/critical disease (serious clinical outcomes, such as ICU admission or death) in patients with COVID-19 pneumonia.

Consistent with previous studies, our study found that age, comorbidities and CT severity score are important and independent predictors of severe/critical or fatality risk of COVID-19 patients.

Age is a pivotal variable in predicting unfavorable outcomes in COVID-19 patients (27, 28). A study of 191 COVID-19 patients showed that older age was associated with a higher risk of in-hospital death, with an odds ratio (OR) of 1.10 for every year added (7). Similarly, Wu and coworkers discovered that older age was related to a higher risk of developing ARDS and dying as a result of it, which is possibly due to elderly patients' lower immune function (27). Older people were considered to be physically frail and more likely to have comorbidities, which placed them at a higher risk of negative consequences, including death (29). Data from the present study support that older age is a predictor for adverse outcomes. In particular, the cut-off point of ~70 years seems to correlate with an augmented risk. Among the possible causes of an increased risk in the elderly population is also considered the treatment with angiotensin-converting enzyme (ACE) inhibitors or angiotensin II type-I receptor blockers (ARBs), drugs that would seem to increase the expression of the ACE-2 receptor, the gateway for the entry of the virus into the cells (30).

Coexisting comorbidities are similarly associated with an increased risk of COVID-19 severity. Huang and colleagues found that a known history of type 2 diabetes increased the likelihood of developing severe illness in COVID-19 patients by six times (31, 32). Another study found that 64.3% of patients had at least one comorbidity, with hypertension (30%) and diabetes (12.1%) being the most common, and that older men with comorbidities were more likely to be affected by COVID-19 (33). Several reports of COVID-19 related comorbidities, on the other hand, proposed that comorbidity should be considered further (32, 34, 35). A more comprehensive evaluation of the impact of various comorbidities on COVID-19 patients is needed and valuable in guiding proper inter-disciplinary management, especially for elderly patients. Patients with severe cardiovascular injury and underlying cardiac insufficiency, for example, are more likely to experience adverse events, according to recent reports (16, 36). For clinical prognosis, summary comorbidity scales such as the CCI are widely used (37, 38). The CCI was created to predict the probability of death within 1 year of being admitted to the hospital. The scores are based on a variety of comorbidities, each of which is assigned a weighted integer from one to six dependent on the severity of the condition (39). CCI is a well-validated and easy-to-use indicator for assessing patient's prognosis and survival. Age, gender, and the existence of comorbidities such as diabetes, cardiovascular, cerebrovascular, and respiratory disorders are frequently used to predict the severity and mortality of COVID-19 during the present pandemic (40–43). In a nationwide study of Danish COVID-19 patients it has been investigated if CCI score was associated with the risk of severe outcome and death (44). It has been found that the odds of severe COVID-19 were significantly increased in CCI score 1–2 (OR 1.7), CCI score 3–4 (OR 2.36), and CCI score >4 (OR 2.67) compared to CCI score 0. In the present study it has been revealed that the CCI score of ≥3 was associated with an ~4-fold time increase in the risk of adverse outcomes. A systematic review and meta-analysis of CCI score and a composite of poor outcomes demonstrated that, compared to a CCI score of 0, a CCI score of 1–2 and CCI score of ≥3 was prognostically associated with mortality and associated with a composite of poor outcomes. Per point increase of CCI score also increased mortality risk by 16%. Moreover, a higher mean CCI score is also significantly associated with mortality and disease severity. The CCI score, which adds together ages and summarizes comorbidity measures, predicts death in COVID-19 patients by an exponential increase in the odds ratio at each point in the score (45).

Lung CT scanning has a higher sensitivity than chest radiography, allowing abnormalities in the lungs to be identified sooner. CT identifies the processes underlying severe/critical conditions and improve clinical diagnosis and treatment by studying their clinical and imaging features. Consistently with several recent reports (46–48), it has been showed that predominant pattern observed was bilateral and peripheral GGO and consolidation (49–51). Despite the fact that pleural and/or pericardial effusion were more frequent in critically ill COVID-19 patients, a multivariable study found no connection between them and death or negative outcomes (10). Qualitative indicators by themselves could differentiate extreme/serious cases from mild/typical cases, but they were unsuccessful in separating severe cases from critical cases. The use of a combination of qualitative and quantitative metrics to identify cases at various clinical stages (52) could assist in the rapid detection and management of critical cases, potentially lowering mortality (19). Previous studies used a semi-quantitative lung CT severity score to measure COVID-19 lung involvement, with the score being assigned based on the severity of all abnormal lung areas involved, similar to the CT severity score system applied to the present study (6, 10, 49, 53, 54). The ideal cut-off point for the CT score employed for study in the prediction of severe/critical disease is 53 (range of the score 0–96).

Conversely, some variables potentially expected to be suggestive of worse outcomes did not prove to be so. For example, cigarette smoking or CRP levels have not been shown to be associated with worse prognosis. High levels of CRP and erythrocyte sedimentation rate (ESR) have been previously reported in COVID-19 patients. Previous works have documented that CRP might play a role in predicting a higher severity of COVID-19 in the early stages, before the development of CT findings (55). A recent systematic review and meta-analysis confirmed that elevated CRP levels are predictors of a worse prognosis in COVID-19 patients (56). Interestingly, a role on CRP values would also be determined by oral cavity health, these two variables being inversely correlated (57).

The main novelty of the present study consists in having applied a comprehensive model of the main variables conditioning outcomes in patients with COVID-19 pneumonia. The integration between the variables was possible through the Fagan nomogram that, calculated on the app, allows a rather rapid estimation of unfavorable prognosis at the patient's bedside. Some works has already proposed the application of algorithms in COVID-19. A retrospective study that assessed the risk of hospitalization in 4,536 COVID-19 patients documented how the variables influencing this risk are multiple, both clinical and demographic (29).

This study has several limitations. First, due to the retrospective nature of the study, there is a chance of knowledge bias. As a result, some information was lacking, and some information, such as the timing of symptom initiation and exposure history, was focused on patients' memories, which may be skewed by recall bias. At the same time, the presence of heterogeneity in some clinical data, such as ICU admission, was unavoidable. Second, there were variations in care, and it was not taken into consideration how treatment influenced prognosis. Third, the data from the two groups were not matched, and the severe/critical group's sample size was limited. Fourth, our patients came from a single geographic area and were treated in a single health system; although our patient population is diverse, factors correlated with poor outcomes may vary elsewhere. Fifth, in this study, well-experienced thoracic radiologists scored the CT images in agreement, and CT scoring is time consuming and can be difficult to apply routinely.

In conclusion, the findings of the present study suggested that the main risk factors predicting a severe/critical outcome in COVID-19 pneumonia patients are older age, comorbidities, and lung CT severity. The prediction model proposed could be used as a quantitative risk predictor method at the time of admission, however it needs further validation in larger cohort studies due to small sample size.

## Data Availability Statement

The raw data supporting the conclusions of this article will be made available by the authors, upon reasonable request to the corresponding author.

## Ethics Statement

The studies involving human participants were reviewed and approved by Review board of Luigi Sacco University Hospital. Written informed consent for participation was not required for this study in accordance with the national legislation and the institutional requirements.

## Author Contributions

FS, PS-P, and MG conceived and designed the study and the protocol. MC, LC, and AG performed the lung CT examinations and their relative interpretation and were involved in revising the paper for important intellectual content. FS and MD carried out data interpretation and analysis. FS, MD, and MC wrote the paper. MC, LC, PS-P, MG, and AG were involved in drafting the article or revising it critically for important intellectual content. All authors have read and approved the final manuscript.

## Conflict of Interest

The authors declare that the research was conducted in the absence of any commercial or financial relationships that could be construed as a potential conflict of interest.

## Publisher's Note

All claims expressed in this article are solely those of the authors and do not necessarily represent those of their affiliated organizations, or those of the publisher, the editors and the reviewers. Any product that may be evaluated in this article, or claim that may be made by its manufacturer, is not guaranteed or endorsed by the publisher.
