# Supplementary material for: Predicting Severe/Critical Outcomes in Patients With SARS-CoV2 Pneumonia: Development of the prediCtion seveRe/crItical ouTcome in COVID-19 (CRITIC) Model
Source: Front Med (Lausanne). 2021 Sep 8;8:695195. doi: 10.3389/fmed.2021.695195 (PMC8456023; doi:10.3389/fmed.2021.695195)
Supplement: Supplementary file 1 [file Table_1.docx]

**Supplementary Table 1.** Criterion values and coordinates of the receiver operating characteristic curve for age.

| Criterion | Sensitivity | 95% CI | Specificity | 95% CI | +LR | -LR |
| --- | --- | --- | --- | --- | --- | --- |
| ≥27.92 | 100.00 | 90.5 - 100.0 | 0.00 | 0.0 - 2.7 | 1.00 | 0.00 |
| >60.06 | 100.00 | 90.5 - 100.0 | 45.93 | 37.3 - 54.7 | 1.85 | 0.00 |
| >60.10 | 97.30 | 85.8 - 99.9 | 45.93 | 37.3 - 54.7 | 1.80 | 0.059 |
| >64.48 | 97.30 | 85.8 - 99.9 | 57.04 | 48.2 - 65.5 | 2.26 | 0.047 |
| >65.00 | 94.59 | 81.8 - 99.3 | 57.04 | 48.2 - 65.5 | 2.20 | 0.095 |
| >68.90* | 94.59 | 81.8 - 99.3 | 68.15 | 59.6 - 75.9 | 2.97 | 0.079 |
| >70.90 | 78.38 | 61.8 - 90.2 | 74.07 | 65.8 - 81.2 | 3.02 | 0.29 |
| >71.33 | 75.68 | 58.8 - 88.2 | 76.30 | 68.2 - 83.2 | 3.19 | 0.32 |
| >72.47 | 67.57 | 50.2 - 82.0 | 79.26 | 71.4 - 85.8 | 3.26 | 0.41 |
| >73.98 | 64.86 | 47.5 - 79.8 | 80.74 | 73.1 - 87.0 | 3.37 | 0.44 |
| >75.90 | 62.16 | 44.8 - 77.5 | 85.19 | 78.1 - 90.7 | 4.20 | 0.44 |
| >78.94 | 40.54 | 24.8 - 57.9 | 93.33 | 87.7 - 96.9 | 6.08 | 0.64 |
| >80.00 | 21.62 | 9.8 - 38.2 | 94.07 | 88.7 - 97.4 | 6.65 | 0.83 |

Abbreviations and legend: CI = confidence interval, +LR = positive likelihood ratio, -LR = negative likelihood ratio, * = optimal cut-off point.

**Supplementary Table 2.** Criterion values and coordinates of the receiver operating characteristic curve for Charlson Comorbidity Index.Criterion values and coordinates of the ROC curve [[Show]](javascript:showdiv('d10','d11','table1');)

| Criterion | Sensitivity | 95% CI | Specificity | 95% CI | +LR | -LR |
| --- | --- | --- | --- | --- | --- | --- |
| >0 | 100.00 | 90.5 - 100.0 | 7.41 | 3.6 - 13.2 | 1.08 | 0.00 |
| >1 | 97.30 | 85.8 - 99.9 | 23.70 | 16.8 - 31.8 | 1.28 | 0.11 |
| >2 | 94.59 | 81.8 - 99.3 | 50.37 | 41.6 - 59.1 | 1.91 | 0.11 |
| >3* | 83.78 | 68.0 - 93.8 | 69.63 | 61.1 - 77.2 | 2.76 | 0.23 |
| >4 | 64.86 | 47.5 - 79.8 | 87.41 | 80.6 - 92.5 | 5.15 | 0.40 |
| >5 | 54.05 | 36.9 - 70.5 | 93.33 | 87.7 - 96.9 | 8.11 | 0.49 |
| >6 | 37.84 | 22.5 - 55.2 | 97.04 | 92.6 - 99.2 | 12.77 | 0.64 |
| >7 | 21.62 | 9.8 - 38.2 | 98.52 | 94.8 - 99.8 | 14.59 | 0.80 |
| >8 | 10.81 | 3.0 - 25.4 | 100.00 | 97.3 - 100.0 | 15.77 | 0.89 |

Abbreviations and legend: CI = confidence interval, +LR = positive likelihood ratio, -LR = negative likelihood ratio, * = optimal cut-off point.

**Supplementary Table 3.** Criterion values and coordinates of the receiver operating characteristic curve for computed tomography severity score.

| Criterion | Sensitivity | 95% CI | Specificity | 95% CI | +LR | -LR |
| --- | --- | --- | --- | --- | --- | --- |
| >23 | 100.00 | 90.5 - 100.0 | 26.67 | 19.4 - 35.0 | 1.36 | 0.00 |
| >24 | 97.30 | 85.8 - 99.9 | 32.59 | 24.8 - 41.2 | 1.44 | 0.083 |
| >25 | 97.30 | 85.8 - 99.9 | 33.33 | 25.5 - 42.0 | 1.46 | 0.081 |
| >28 | 94.59 | 81.8 - 99.3 | 37.78 | 29.6 - 46.5 | 1.52 | 0.14 |
| >30 | 89.19 | 74.6 - 97.0 | 41.48 | 33.1 - 50.3 | 1.55 | 0.26 |
| >36 | 86.49 | 71.2 - 95.5 | 59.26 | 50.5 - 67.6 | 2.12 | 0.23 |
| >38 | 86.49 | 71.2 - 95.5 | 62.22 | 53.5 - 70.4 | 2.29 | 0.22 |
| >40 | 78.38 | 61.8 - 90.2 | 64.44 | 55.8 - 72.5 | 2.40 | 0.34 |
| >43 | 78.38 | 61.8 - 90.2 | 68.89 | 60.4 - 76.6 | 2.52 | 0.31 |
| >45 | 72.97 | 55.9 - 86.2 | 71.11 | 62.7 - 78.6 | 2.53 | 0.38 |
| >46 | 72.97 | 55.9 - 86.2 | 72.59 | 64.3 - 79.9 | 2.66 | 0.37 |
| >48 | 67.57 | 50.2 - 82.0 | 77.78 | 69.8 - 84.5 | 3.04 | 0.42 |
| >49 | 64.86 | 47.5 - 79.8 | 77.78 | 69.8 - 84.5 | 3.12 | 0.45 |
| >53* | 64.86 | 47.5 - 79.8 | 84.44 | 77.2 - 90.1 | 4.17 | 0.42 |
| >54 | 59.46 | 42.1 - 75.2 | 87.41 | 80.6 - 92.5 | 4.72 | 0.46 |
| >55 | 56.76 | 39.5 - 72.9 | 88.89 | 82.3 - 93.6 | 5.11 | 0.49 |
| >64 | 51.35 | 34.4 - 68.1 | 93.33 | 87.7 - 96.9 | 7.70 | 0.52 |
| >68 | 37.84 | 22.5 - 55.2 | 95.56 | 90.6 - 98.4 | 8.51 | 0.65 |
| >71 | 32.43 | 18.0 - 49.8 | 96.30 | 91.6 - 98.8 | 8.76 | 0.70 |
| >73 | 32.43 | 18.0 - 49.8 | 99.26 | 95.9 - 100.0 | 40.78 | 0.68 |
| >77 | 24.32 | 11.8 - 41.2 | 99.26 | 95.9 - 100.0 | 42.84 | 0.76 |

Abbreviations and legend: CI = confidence interval, +LR = positive likelihood ratio, -LR = negative likelihood ratio, * = optimal cut-off point.
